# Monitoring gamma type-I censored data using an exponentially weighted moving average control chart based on deep learning networks

**DOI:** 10.1038/s41598-024-56884-8

**Published:** 2024-03-18

**Authors:** Pei-Hsi Lee, Shih-Lung Liao

**Affiliations:** Department of Information Management, Chaoyang University of Technology, Taichung, Taiwan

**Keywords:** Deep learning methods, Conditional expected value, Conditional median, Right-censored data, Exponentially weighted moving average chart, Computer science, Statistics

## Abstract

In recent years, deep learning methods have been widely used in combination with control charts to improve the monitoring efficiency of complete data. However, due to time and cost constraints, data obtained from reliability life tests are often type-I right censored. Traditional control charts become inefficient for monitoring this type of data. Thus, researchers have proposed various control charts with conditional expected values (CEV) or conditional median (CM) to improve efficiency for right-censored data under normal and non-normal conditions. This study combines the exponentially weighted moving average (EWMA) CEV and CM chart with deep learning methods to increase efficiency for gamma type-I right-censored data. A statistical simulation and a real-world case are presented to assess the proposed method, which outperforms the traditional EWMA charts with CEV and CM in various skewness coefficient values and censoring rates for gamma type-I right-censored data.

## Introduction

In recent years, artificial-intelligence technology has been widely used in production processes to reduce labor and quickly respond to process variations. In particular, machine learning and deep learning methods have been introduced into process control and excelled in quickly detecting and correctly identifying abnormal process conditions. For example, Lee and Kim^1^, Wang et al.^2^, Chen and Yu^3^, Kim and Ha^4^, Yeganeh et al.^5^, and Sabahno and Amiri^6^ proposed control charts based on machine or deep learning to improve detection ability. Zhang et al.^7^, Yu and Liu^8^, Maged et al.^9^, and Yu et al.^10,11^ achieved excellent performance in failure detection in high-dimensional or complex processes using deep learning methods. Moreover, Zan et al.^12^, Lu et al.^13^, Yu and Zhang^14^, Lee et al.^15^, and Xue et al.^16^ used machine and deep learning methods to recognize abnormal control chart patterns.

To test the lifetime of electronic products, practitioners may set a test termination time at which to halt the test in order to reduce time and cost. As some tested units may not have failed by the termination time, practitioners can only obtain incomplete lifetime records, which are known as type-I right-censored data. This is a challenge for practitioners because the detection efficiency for process variation of traditional Shewhart-type and EWMA-type control charts decrease for incomplete data. Thus, some researchers have proposed novel control charts to monitor right-censored data^10,11,17,18^. Steiner and Mackay^19^ estimated the mean lifetime of incomplete data with a CEV and combined it with an $\overline{X}$ chart of a lower control limit (LCL) to detect decreases of the lifetime for highly right-censored data with assumptions of normality. Then, Steiner and Mackay^20^ used Shewhart-type CEV control charts for censored data with non-normality conditions. They next Steiner and Mackay^21^ combined the EWMA and CEV to increase the detection ability for highly right-censored data. Lee^22^ determined the design parameters of the CEV $\overline{X}$ control chart with a minimum cost perspective.

Zhang and Chen^23^ assumed a Weibull distribution for the data and detected decreases and increases in the average lifetime for censored data using two single-sided EWMA CEV control charts. Tsai and Lin^24^, Raza et al.^25^, and Biozone and Wang^26^ also developed EWMA-type CEV control charts and achieved good monitoring performance in non-normal censored data for their proposed charts. Raza et al.^27^ and Raza and Siddiqi^28^ established a double EWMA (DEWMA) control chart to detect shifts of the scale parameter for gamma censored data. Raza and Siddiqi^28^, Raza et al.^29^, and Ali et al.^30^ proposed similar contributions to the DEWMA CEV control chart. Ali et al.^31^ and Ahmed et al.^32,33^ developed novel control charts to monitor Weibull and generalized exponential (GE) censored data by replacing CEV with CM and conditional standard deviation (CSD). In addition, Lee et al.^34^ and Zhao and Wu^35^ implemented the EWMA CEV chart for multiple censored data and window-censored data, respectively. However, to the authors’ knowledge, there have been no relevant studies monitoring right-censored data using deep learning methods.

Convolutional neural networks (CNN) and long short-term memory (LSTM) networks are common methods of deep learning that have been successfully combined with control chart technology to monitor processes and recognize abnormal patterns in control charts^7,14,36^. Thus, these techniques may be used to improve the efficiency of a control chart for censored data. From the literature, most previous works developed CEV and CM charts for normal, GE or Weibull censored data. Gamma data is also often observed in reliability life tests and is one of the commonly used lifetime distributions because changing its shape parameter can create distributions with different degrees of skewness. Therefore, this study combines a CNN and LSTM, respectively, with the EWMA CEV and CM charts to monitor gamma type-I right-censored data.

The remainder of this study is organized as follows. In “Methodology” section presents the methodology. In “Proposed control charts” section describes the proposed control chart and in “Performance comparison” section investigates its performance using a statistical simulation. In “Real-world case study” section details implementation of the proposed method for a case study. Finally, in “Conclusions” section provides some conclusions and directions for future development.

## Methodology

### EWMA charts for gamma type-I censored data

Let $U = \left\{ {u_{1} ,u_{2} , \ldots ,u_{m} } \right\}$ be a gamma random variable and $a_{0}$ and $b_{0}$ be the shape and scale parameters of an in-control process, respectively. The censoring rate for the gamma lifetimes can be represented as $Pc = 1 - F_{Ga} \left( {u = c_{T} {|}a_{0} ,b_{0} } \right)$, where $F_{Ga} \left( { \cdot {|}a_{0} ,b_{0} } \right)$ is the cumulative distribution function (CDF) of the gamma distribution with parameters $a_{0}$ and $b_{0}$, where $c_{T}$ is the censoring time. The CEV of the gamma distribution is:

1 $$Cev = E\left( {U{|}u \ge c_{T} } \right) = \frac{{a_{0} b_{0} \left[ {1 - F_{Ga} \left( {u = c_{T} {|}a_{0} + 1,b_{0} } \right)} \right]}}{{1 - F_{Ga} \left( {u = c_{T} {|}a_{0} ,b_{0} } \right)}},$$

and the CM of the gamma distribution is

2 $$CM = F_{Ga}^{ - 1} \left( {0.5 - 0.5F_{Ga} \left( {u = c_{T} {|}a_{0} ,b_{0} } \right){|}a_{0} ,b_{0} } \right),$$

where $F_{Ga}^{ - 1} \left( { \cdot {|}a_{0} ,b_{0} } \right)$ is an inverse of CDF of the gamma distribution with $a_{0}$ and $b_{0}$. The derivations of Eqs. (1) and (2) are shown in Supplementary. Practitioners take $n$ samples and measure their lifetime values using the reliability life testing method. The sample mean of size $n$ can be obtained by $\overline{X} = \sum\nolimits_{i = 1}^{n} {x_{i} } /n$. Let $u_{i}$ be the lifetime of the *i*-th testing sample. Then, the $x_{i}$ of the *i*-th testing sample in the $\overline{X}$’s formula is:

3 $$x_{i} = \left\{ {\begin{array}{*{20}l} {u_{i} } \hfill & {\quad for\;u_{i} \le c_{T} } \hfill \\ {Cd} \hfill & {\quad for\;u_{i} > c_{T} } \hfill \\ \end{array} } \right.,$$

where $Cd = Cev$ for CEV $\overline{X}$ statistic or $CM$ for CM $\overline{X}$ statistic. The in-control mean $M_{0}$ and variance $V_{0}$ can be expressed as follows:

$$\begin{aligned} & M_{0} = a_{0} b_{0} \;\;{\text{and}} \\ & V_{0} = a_{0} b_{0}^{2} . \\ \end{aligned}$$

Let $\lambda$ be the smoothing parameter of the EWMA chart. Zhang and Chen^23^ showed that the EWMA statistic at period *j* for monitoring the mean decrease is:

4 $$E_{j} = min\left\{ {M_{0} ,\lambda \overline{{X_{j} }} + \left( {1 - \lambda } \right)E_{j - 1} } \right\}.$$

For an EWMA chart with CEV or CM used to monitor a process mean, an LCL is set to signal the mean reduction because practitioners always focus on the detection of average lifetime reduction. The appearance of an assignable cause leads to a decrease in the process mean, indicating an out-of-control condition. Let $M_{1} = \delta \times M_{0}$ be the mean of an out-of-control state where the process variance is unchanged and $\delta$ be a mean shift size that can be obtained by $\delta = M_{1} /M_{0}$. The gamma shape parameter $a_{1}$ and scale parameter $b_{1}$ in an out-of-control state can be obtained by solving the following system of simultaneous equations:

$$\left\{ {\begin{array}{*{20}l} {M_{1} = a_{1} b_{1} } \hfill \\ {V_{0} = a_{1} b_{1}^{2} } \hfill \\ \end{array} } \right.,$$

The solutions for $a_{1}$ and $b_{1}$ are, respectively, as follows:

5 $$\begin{aligned} a_{1} & = M_{1}^{2} /V_{0} \;\;{\text{and}} \\ b_{1} & = V_{0} /M_{1} . \\ \end{aligned}$$

The primary metric employed for evaluating the effectiveness of control charts is the average run length (ARL)^3,15,23,30,34^. In an in-control process, a larger ARL signifies a reduced false-alarm rate, while in an out-of-control state, a smaller ARL indicates quicker detection of mean reduction.

### CNN

The main advantage of CNNs is that they can effectively capture local features in the data and perform feature extraction for classification or regression prediction, while maintaining the spatial hierarchy of features. Some of the main features and working principles of CNN are laid out below.

The convolutional layer is a fundamental element of CNNs. It operates by applying convolutional kernels (also referred to as filters) over the input data or image through a sliding process, resulting in the generation of feature maps. This operation supports the identification of local features. The output of a convolutional layer $l$ can be expressed by:

$$\zeta_{l}^{CL} = f_{RL} \left( {b_{l}^{CL} + \zeta_{l - 1}^{CL} \times \omega_{l - 1}^{CL} } \right),$$

where $f_{RL} \left( \cdot \right)$ is a rectified linear unit (ReLU) activation function, $l$ is the $l$-th layer of the CNN, $\omega_{l - 1}^{CL}$ is the filter kernel at layer $l - 1$, and $b_{l}^{CL}$ is the bias vector at layer $l$.

The pooling layer plays a crucial role in CNNs by decreasing the dimensionality of feature maps while retaining vital information. The most common pooling operation is max pooling, which selects the highest value within specific regions, thus reducing the feature map’s dimensions. The pooling output at layer $l$ is given by:

$$Mp_{j} = max\left( {\zeta_{l}^{CL} } \right).$$

After the convolutional and pooling layers, CNNs frequently incorporate fully connected layers to carry out ultimate classification or regression tasks. These layers are responsible for transforming the extracted features into the network’s final output. CNNs commonly comprise a series of convolutional and pooling layers stacked in an interleaved fashion. This layered architecture empowers the network to acquire knowledge about image characteristics spanning diverse levels of abstraction^7,8^.

### LSTM

An LSTM is a deep learning neural network architecture that is an improvement of over traditional recurrent neural networks (RNN). It was specially designed to process sequence data, such as speech recognition, natural language processing, time-series analysis, and other applications. LSTM supports sequence data processing by introducing the three key gating mechanisms described below.

The forget gate determines whether to forget the previous memory information. It uses a sigmoid function to output a value between 0 and 1, controlling whether past memories are retained. The forget gate ${f}^{fg}$ can be expressed as:

$$f^{fg} = f_{sf} \left( {b^{fg} + \omega^{fg} \left[ {\zeta_{t - 1} ,\vartheta_{t} } \right]} \right),$$

where $f_{sf} \left( \cdot \right)$ is a sigmoid function, $b^{fg}$ is the bias vector of the forget gate, $\omega^{fg}$ is the weight vector of the forget gate, $\zeta_{t - 1}$ is the output vector of the previous step, and $\vartheta_{t}$ is the input vector of the current step.

The input gate determines how new memory information is added to the LSTM unit. It uses a sigmoid function to determine which information needs updating and uses the tanh function to create a memory cell vector. Let $f^{ig}$ be the formula of the input gate and $\epsilon_{t}$ be the memory cell vector of the current step, as follows:

$$\begin{aligned} & f^{ig} = f_{sf} \left( {b^{ig} + \omega^{ig} \left[ {\zeta_{t - 1} ,\vartheta_{t} } \right]} \right)\;\;{\text{and}} \\ & \epsilon_{t} = f^{fg} \times \epsilon_{t - 1} + f^{ig} \times tanh\left( {b^{\epsilon} + \omega^{\epsilon} \left[ {\zeta_{t - 1} ,\vartheta_{t} } \right]} \right), \\ \end{aligned}$$

where $b^{ig}$ is the bias vector of the input gate, $\omega^{ig}$ is the weight vector of the input gate, $tanh\left( \cdot \right)$ is the tanh function, and $b^{\epsilon}$ and $\omega^{\epsilon}$ are the bias and weight vectors of the memory cell vector for the current step, respectively.

The output gate determines which memory information will be outputted to the next time step. Similar to the forget gate and input gate, the output gate uses a sigmoid function to control the output. The output gate $f^{og}$ is:

$$f^{og} = f_{sf} \left( {b^{og} + \omega^{og} \left[ {\zeta_{t - 1} ,\vartheta_{t} } \right]} \right),$$

where $b^{og}$ is the bias vector of the output gate and $\omega^{og}$ is the weight vector of the output gate. The output vector of the current step $\zeta_{t}$ is:

$$\zeta_{t} = f^{og} \times tanh\left( {\epsilon_{t} } \right).$$

The dimensions of $\zeta_{t}$ as the number of hidden units affects the LSTM network’s computing efficiency and effectiveness. LSTM uses these gates to control the flow of information and update memory, thus helping to address the gradient vanishing problem in RNNs and allowing them to better handle long sequences^9,36^.

## Proposed control charts

**Figure 1:**
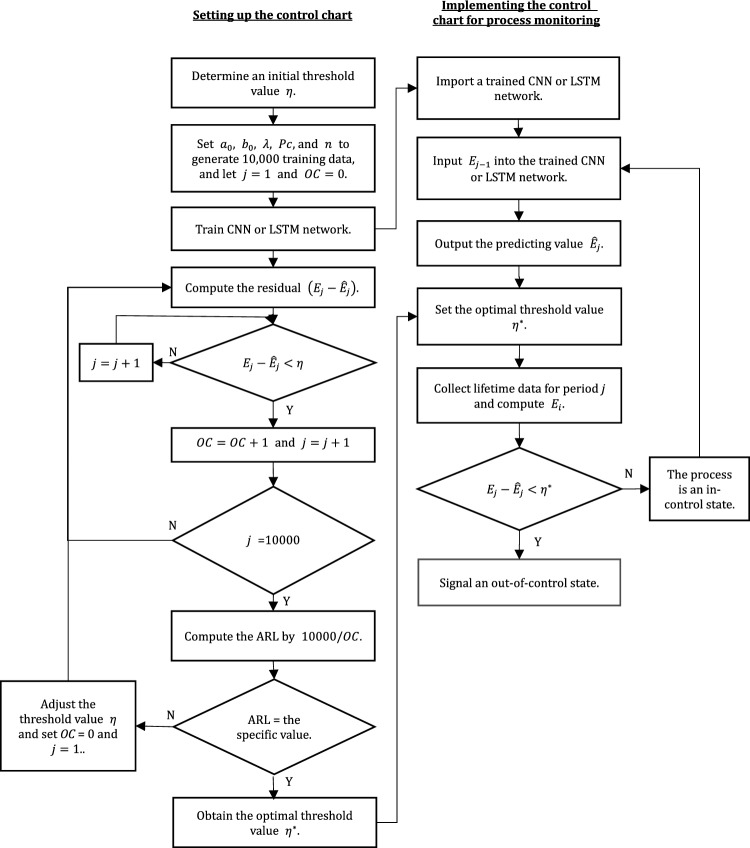
Flowchart for the proposed control chart.

This section proposes a procedure to set and implement the control chart based on deep learning networks with EWMA CEV or CM statistic, as shown in Fig. 1. This procedure includes two parts: setting up the control chart and implementing the control chart.

The purpose of setting up the control chart is to find the optimal threshold value according to a specific in-control ARL value to maintain the monitoring effect of the control chart. Before setting up a control chart, practitioners can determine an in-control ARL value and choose an initial threshold value based on their own experience. In the literature, the in-control ARL value used for control chart performance comparison is typically 200 or 370.4.

According to the quality characteristic parameters and control parameters such as $a_{0}$, $b_{0}$, $\lambda$, $Pc$, and $n$, the in-control gamma type-I censored data can be generated using the Monte Carlo method and then the in-control EWMA statistics $E_{j}$ for the gamma type-I censored data can be calculated using Eq. (4). It is noted that $E_{j}$ is the EWMA CEV statistic for $Cd = Cev$ or the EWMA CM statistic for $Cd = CM$. First, 10,000 $E_{j}$ are generated. Next, let $\left\{ {E_{t - 1} ,E_{t} } \right\}$ be the training data set and be inputted into the CNN or LSTM network for training. As the training data set, the input layer of the CNN or LSTM network must use one-dimensional sequence data. The input and output vectors have dimensions $1 \times \left( {m - 1} \right)$.

Practitioners are more concerned about decreases in the average lifetime, so the threshold value $\eta$ as a LCL of traditional control charts is set to detect such decreases. After the network is trained, the estimation $\widehat{{E_{t} }}$ can be outputted and the residual value can be computed by $E_{t} - \widehat{{E_{t} }}$. If the *j*-th residual value is less than the threshold value $\eta$, then the number of points outside the threshold value (*OC*) = *OC* + 1. The above procedure is repeated 10,000 times to obtain total number of points outside the threshold value and the in-control ARL value is 10,000/*OC* (Note that the false alarm rate is *OC*/10,000).

If the simulated in-control ARL value is not equal to the specific in-control ARL value, then the threshold value $\eta$ is adjusted and the above simulation procedure is repeated until the simulated ARL value equals the specific ARL value. In this way, the optimal threshold value $\eta^{*}$ can be obtained and implemented to monitor the process.

In the implementation of the control chart for process monitoring, practitioners import a trained CNN or LSTM network, set up the optimal threshold value $\eta^{*}$, and then apply the following steps:

1. Collect lifetime data for period *t* − 1 and calculate the statistic $E_{t - 1}$.
2. Input $E_{t - 1}$ into this trained network to predict the statistic $\widehat{{E_{t} }}$ of period *t*.
3. After the lifetime data for period *t* is obtained, compute the actual value $E_{t}$.
4. Let the error value for period* t* be $E_{t} - \widehat{{E_{t} }}$.

If the error value is less than the optimal threshold value $\eta^{*}$, then the process indicates an out-of-control condition; otherwise, the process is in control.

**Figure 2:**
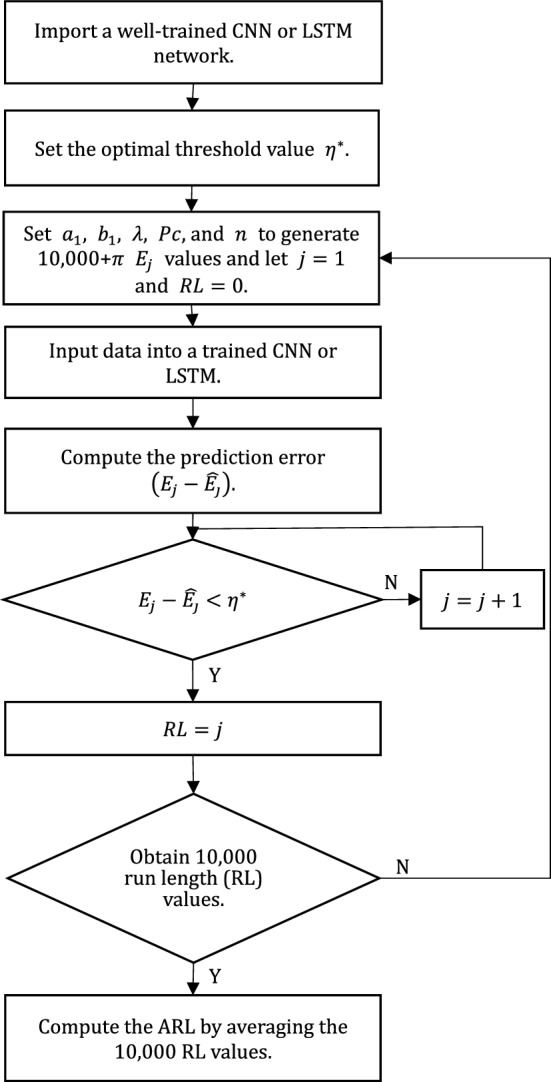
Flowchart for simulation of an ARL value using the proposed control chart.

Figure 2 shows the simulation process for an out-of-control ARL value. Practitioners can give a shift size value $\delta$ to calculate the parameters $a_{1}$ and $b_{1}$ of the out-of-control state shown in Fig. 2 using Eq. (5).

Some studies typically considered two different types of performance: zero-state (ZS) and steady-state (SS)^37,38^. ZS performance assumes that a shift occurs at the beginning of the process to measure the out-of-control ARL value. SS performance shows the out-of-control ARL for control charts to identify a process shift for control statistics to reach a static distribution.

For the simulated data generation of ZS and SS conditions, assume that the process has been continuously run for $\pi$ sampling periods and maintained in the in-control state, $\pi$ represents the length of the process to reach SS condition. The process occurs the mean shift between $\pi$th and $\pi$ + 1st sampling, and then SS ARL represents the expected value of the number of samples obtained from the occurrence of this mean shift to when the chart indicates an out-of-control signal. In the data generation process, the in-control data of $\pi$ periods is first generated. After the in-control data, 10,000 out-of-control data are generated. The EWMA statistics are calculated for the data of $\pi$ + 10,000 using Eq. (4), and the last 10,000 EWMA statistics are taken to simulate the out-of-control ARL values of SS condition according to Fig. 2. For ZS condition, the simulated data can be generated with a setting of $\pi = 0$.

MATLAB R2023a provides a deep learning toolbox that can easily implement the processes of Figs. 1 and 2. This study codes the simulation processes using MATLAB R2023a to investigate the performance of the proposed control charts.

## Performance comparison

Hereafter, ‘CNN chart’ and ‘LSTM chart’ represent the control charts based on the CNN and LSTM networks, respectively. This section compares the ARL performance of the CNN, LSTM, and EWMA charts with CEV or CM for gamma type-I censored data. The parameters of the gamma distribution are set as $a_{0}$ = 1, 2, and 4 and $b_{0} = 1$ for comparison, while the skewness values (Sk) of the parameter case are as shown in Table 1. Smaller $a_{0}$ values indicate greater skewness of the gamma distribution.

**Table 1:** Skewness values of the gamma distribution.

$a_{0}$	$b_{0}$	$M_{0}$	$V_{0}$	SK
1	1	1	1	2
2	1	2	2	1.4142
4	1	4	4	1

When using the CNN network, some network parameters, such as the stacked numbers of convolutional and pooling layers, kernel size, and number of kernels, must be determined first to achieve good training and testing results. For the LSTM chart, the stacked numbers of LSTM layers and the number of hidden units must also be decided. Based on the literature, this study uses the trial-and-error method to set these network parameters as per Table 2^3,39^.

**Table 2:** Structure and parameters of deep learning networks for the proposed control charts.

Layers	CNN				LSTM	
	Input layer	Convolutional layer		Pooling layer	Input layer	Number of hidden units
		Kernel size	Number of kernels	Kernel size		
#1	One-dimensional sequence data	5	200	1	One-dimensional sequence data	100
#2	–	3	70	1	–	70
#3	–	3	50	1	–	50
#4	–	3	40	1	–	40
#5	–	3	40	1	–	30
#6–24	–	3	40	1	–	–
#25–36	–	3	30	1	–	–

In the performance comparison, the sample size is fixed at $n = 5$, which is standard practice for sampling and plotting control charts. The smoothing parameter $\lambda$ of the EWMA statistic is set at 0.1 and 0.2^23,27,29^. $Pc$ is 0.2, 0.5, and 0.8 for lower, moderate, and higher censoring rates, respectively. The shift sizes are $\delta = 0.8$ and 0.7 for small shifts; $\delta = 0.6$ and 0.5 for moderate shifts; and $\delta = 0.2$ for large shifts. This study set the in-control ARL value at 200 to measure the out-of-control ARL values under ZS and SS conditions.

Considering that different trained CNN or LSTM networks will have different ARL values, this study trained 100 networks for each condition of process parameters ($a_{0}$, $b_{0}$, $Pc$ and $\lambda$) and then selected a trained network with the smallest ARL value for comparison from the 100 trained networks.

Table 3 shows the LCL and the optimal threshold value $\eta^{*}$ for the six comparison charts under ZS condition ($\pi$ = 0). The out-of-control ARL values for the six control charts were simulated according to the above conditions and the ARL values are compared in Table 4. The bold cells indicate the control chart with the best detection efficiency for a specific shift size.

**Table 3:** Design parameter values of six control charts for comparison under ZS condition.

($a_{0}$, $b_{0}$)	$Pc$	*λ* = 0.1						*λ* = 0.2					
		EWMA		CNN		LSTM		EWMA		CNN		LSTM	
		LCL		$\eta^{*}$		$\eta^{*}$		LCL		$\eta^{*}$		$\eta^{*}$	
		CEV	CM	CEV	CM	CEV	CM	CEV	CM	CEV	CM	CEV	CM
(1, 1)	0.2	0.793	0.370	− 0.071	− 0.047	− 0.170	− 0.107	0.685	0.358	− 0.090	− 0.037	− 0.238	− 0.145
	0.5	0.828	0.269	− 0.062	− 0.011	− 0.134	− 0.032	0.732	0.251	− 0.057	− 0.013	− 0.222	− 0.052
	0.8	0.885	0.394	− 0.054	− 0.022	− 0.104	− 0.045	0.815	0.368	− 0.045	− 0.022	− 0.165	− 0.072
(2, 1)	0.2	1.695	1.101	− 0.083	− 0.060	− 0.284	− 0.193	1.535	1.005	− 0.090	− 0.068	− 0.391	− 0.279
	0.5	1.729	0.891	− 0.163	− 0.035	− 0.250	− 0.071	1.585	0.849	− 0.124	− 0.031	− 0.325	− 0.123
	0.8	1.805	1.124	− 0.074	− 0.053	− 0.182	− 0.101	1.691	1.067	− 0.098	− 0.055	− 0.261	− 0.151
(4, 1)	0.2	3.550	2.409	− 0.181	− 0.149	− 0.409	− 0.321	3.315	2.515	− 0.186	− 0.118	− 0.569	− 0.447
	0.5	3.600	2.381	− 0.276	− 0.095	− 0.344	− 0.149	3.374	2.298	− 0.194	− 0.061	− 0.506	− 0.225
	0.8	3.697	2.757	− 0.149	− 0.206	− 0.297	− 0.186	3.507	2.655	− 0.189	− 0.167	− 0.414	− 0.151

**Table 4:** ZS ARL values of six control charts.

$Pc$	$\delta$	($a_{0}$, $b_{0}$) = (1, 1)						($a_{0}$, $b_{0}$) = (2, 1)						($a_{0}$, $b_{0}$) = (4, 1)					
		EWMA		CNN		LSTM		EWMA		CNN		LSTM		EWMA		CNN		LSTM	
		CEV	CM	CEV	CM	CEV	CM	CEV	CM	CEV	CM	CEV	CM	CEV	CM	CEV	CM	CEV	CM
$$\lambda = 0.1$$																			
0.2	0.8	16.18	32.43	4.64	**1.62**	39.13	40.44	11.67	27.30	**1.94**	3.99	25.00	31.29	8.19	21.76	**2.13**	7.00	12.70	20.45
	0.7	9.61	22.69	1.99	**1.52**	26.32	21.61	6.89	18.38	1.65	**1.39**	15.42	19.09	5.11	14.26	**1.42**	2.12	8.67	13.16
	0.6	6.82	17.55	**1.30**	1.38	21.51	17.53	5.08	13.75	**1.18**	1.30	13.41	14.87	3.75	10.56	**1.15**	1.27	8.39	9.66
	0.5	5.24	14.31	**1.22**	1.21	18.58	14.25	3.91	10.98	**1.11**	1.18	12.56	12.32	2.98	8.31	1.13	**1.05**	7.49	7.98
	0.2	3.22	9.76	**1.00**	1.02	13.04	9.43	2.41	7.04	**1.00**	**1.00**	9.14	8.46	2.05	5.10	**1.00**	**1.00**	6.50	5.80
0.5	0.8	13.36	27.70	**2.15**	3.26	29.83	19.06	10.49	29.77	1.81	**1.27**	20.05	21.53	7.50	23.84	**2.08**	3.66	11.56	21.81
	0.7	7.66	23.17	**1.55**	1.72	21.92	15.97	6.26	21.91	1.30	**1.10**	13.52	17.86	4.55	16.85	**1.30**	1.78	10.15	14.22
	0.6	5.39	20.00	**1.37**	1.51	19.38	14.16	4.45	17.25	1.19	**1.01**	12.43	13.70	3.37	13.04	**1.14**	1.32	8.78	10.04
	0.5	4.16	17.46	1.24	**1.19**	16.30	12.68	3.45	14.21	1.13	**1.00**	9.56	13.22	2.69	10.49	**1.03**	1.06	8.88	9.46
	0.2	2.50	12.99	1.01	**1.00**	13.02	9.45	2.16	9.32	**1.00**	**1.00**	8.81	9.53	2.01	6.44	**1.00**	**1.00**	6.43	6.22
0.8	0.8	10.77	28.75	6.73	**5.02**	31.94	22.12	8.90	23.07	3.71	**2.32**	19.29	23.17	6.95	18.18	**4.72**	5.19	10.61	19.95
	0.7	5.89	21.81	2.71	**2.42**	22.89	17.77	5.32	16.82	1.70	**1.69**	13.55	16.86	4.11	12.73	**2.19**	2.43	9.43	14.73
	0.6	4.10	17.56	1.57	**1.29**	18.49	15.60	3.68	12.96	**1.24**	1.37	11.61	13.73	2.87	9.62	**1.19**	1.66	8.10	10.75
	0.5	3.10	14.71	1.29	**1.02**	16.28	12.57	2.78	10.57	**1.08**	1.24	10.31	11.45	2.25	7.72	**1.07**	1.32	8.23	9.81
	0.2	2.02	10.29	**1.00**	**1.00**	11.75	9.18	1.66	6.86	**1.00**	**1.00**	7.79	7.32	1.34	4.81	**1.00**	**1.00**	5.71	5.12
$$\lambda = 0.2$$																			
0.2	0.8	16.85	25.88	9.36	**3.55**	45.41	49.78	10.88	20.59	**1.92**	3.51	21.09	25.08	7.28	16.00	**1.69**	2.14	13.77	19.04
	0.7	8.83	15.27	4.91	**2.57**	30.87	23.03	6.52	12.12	**1.50**	1.83	16.00	15.98	4.24	9.19	**1.31**	1.49	8.61	11.73
	0.6	6.04	10.92	2.97	**1.95**	22.21	17.56	4.28	8.42	**1.26**	1.38	12.89	11.58	3.19	6.45	**1.13**	1.20	8.28	9.15
	0.5	4.38	8.61	2.41	**1.75**	20.44	11.19	3.24	6.57	**1.13**	1.15	11.77	10.90	2.51	4.98	**1.04**	1.05	7.55	7.33
	0.2	2.46	5.58	**1.24**	**1.24**	12.32	6.98	2.09	4.12	**1.01**	**1.01**	9.11	6.96	1.49	3.08	**1.00**	**1.00**	5.51	3.58
0.5	0.8	14.04	22.52	7.95	**3.07**	44.87	19.44	10.18	18.25	3.12	**2.34**	25.09	26.28	7.19	14.24	2.41	**1.81**	12.97	13.35
	0.7	7.22	16.01	3.80	**1.69**	31.69	12.49	5.49	12.31	1.94	**1.84**	17.20	19.13	4.13	9.60	**1.36**	1.43	8.98	8.47
	0.6	4.94	12.42	2.45	**1.19**	24.57	11.60	3.78	9.39	1.57	**1.32**	14.62	14.10	2.89	7.14	**1.12**	1.15	6.68	7.88
	0.5	3.57	10.45	1.63	**1.07**	21.10	8.43	2.88	7.62	1.37	**1.19**	12.46	11.53	2.28	5.69	**1.05**	1.07	6.84	5.38
	0.2	2.15	7.27	1.08	**1.00**	11.75	5.82	2.04	5.00	**1.01**	**1.01**	10.18	5.86	1.35	3.48	**1.00**	**1.00**	5.99	4.74
0.8	0.8	10.61	18.33	3.04	**2.74**	30.25	26.39	8.86	14.57	**2.55**	3.28	21.92	20.53	6.67	11.37	**1.85**	1.95	12.29	18.02
	0.7	5.60	12.53	2.06	**1.67**	22.08	16.47	4.49	9.57	**1.74**	**1.74**	14.80	14.01	3.72	7.39	**1.11**	1.34	8.61	12.52
	0.6	3.62	9.70	1.95	**1.33**	18.26	12.95	3.06	7.34	1.49	**1.22**	12.37	10.95	2.54	5.55	**1.02**	1.18	7.46	10.55
	0.5	2.65	8.10	1.50	**1.09**	14.42	9.31	2.35	5.93	1.32	**1.05**	11.02	10.20	1.94	4.41	**1.00**	1.10	7.38	7.32
	0.2	1.43	5.45	1.03	**1.00**	10.36	5.41	1.46	3.87	**1.00**	**1.00**	9.18	5.74	1.10	2.84	**1.00**	**1.00**	6.15	4.63

Smallest ARL values are in [bold].

As the skewness coefficient value decreases, the detection efficiency of the six control charts decreases. As $Pc$ increases, the detection efficiency of the EWMA chart decreases, and the detection efficiency of the CNN and LSTM charts changes irregularly. The CNN chart exhibits the best detection ability for most shift sizes. The LSTM chart is significantly worse than the other charts for all shift sizes. Comparing the performance of CEV and CM, CNN with CEV is better than CNN with CM when the skew coefficient value is small. As the skew coefficient value becomes larger, the effect of CNN with CM is better than CNN with CEV. EWMA and LSTM charts are not affected by the skewness coefficient. EWMA and LSTM charts with CEV has better performance than EWMA and LSTM charts with CM.

For processes that often occur the mean shift in the initial stage, if the skewness coefficient of lifetime distribution is large, the EWMA CM statistic should be used to train the CNN network and implement monitoring. On the contrary, the EWMA CEV statistic should be considered to train the CNN network and implement monitoring.

This study considers $\pi$ = 100 and 1000 to measure the ARL value of SS condition. Table 5 shows the LCL and $\eta^{*}$ of six control charts for $\pi$ = 1000. Table 7 exhibits the SS ARL values for $\pi$ = 100 and 1000, respectively.

**Table 5:** Design parameter values of six control charts for comparison under SS condition.

($a_{0}$, $b_{0}$)	$Pc$	*λ* = 0.1						*λ* = 0.2					
		EWMA		CNN		LSTM		EWMA		CNN		LSTM	
		LCL		$\eta^{*}$		$\eta^{*}$		LCL		$\eta^{*}$		$\eta^{*}$	
		CEV	CM	CEV	CM	CEV	CM	CEV	CM	CEV	CM	CEV	CM
(1, 1)	0.2	0.784	0.406	− 0.111	− 0.034	− 0.807	− 0.399	0.685	0.356	− 0.064	− 0.047	− 0.795	− 0.398
	0.5	0.827	0.268	− 0.057	− 0.018	− 0.799	− 0.154	0.732	0.250	− 0.062	− 0.015	− 0.789	− 0.151
	0.8	0.876	0.392	− 0.056	− 0.025	− 0.649	− 0.213	0.816	0.367	− 0.052	− 0.012	− 0.662	− 0.223
(2, 1)	0.2	1.689	1.096	− 0.118	− 0.053	− 1.229	− 0.756	1.531	1.001	− 0.169	− 0.053	− 1.266	− 0.745
	0.5	1.733	0.888	− 0.122	− 0.031	− 1.234	− 0.343	1.579	0.847	− 0.137	− 0.042	− 1.238	− 0.377
	0.8	1.802	1.120	− 0.103	− 0.052	− 1.099	− 0.508	1.690	1.065	− 0.076	− 0.047	− 1.098	− 0.498
(4, 1)	0.2	3.546	2.662	− 0.111	− 0.160	− 1.236	− 1.236	3.314	2.511	− 0.285	− 0.139	− 1.265	− 1.265
	0.5	3.595	2.373	− 0.127	− 0.060	− 1.910	− 0.732	3.372	2.295	− 0.192	− 0.085	− 0.731	− 1.872
	0.8	3.690	2.750	− 0.135	− 0.041	− 1.640	− 0.213	3.507	2.649	− 0.154	− 0.095	− 1.655	− 0.915

As Table 6, CNN chart outperforms EWMA and LSTM for most shift sizes in the $\pi$ = 1000. With only some cases of $\delta$ = 0.8, the EWMA chart is better than the CNN chart. LSTM chart in most cases of $\delta$ ≤ 0.5 has better detection efficiency than EWMA chart, but it's still not as efficient as CNN chart. The detection efficiency of LSTM charts in other shift sizes is worse than that of EWMA and CNN charts. The detection efficiency of the EWMA, CNN and LSTM charts decreases as $Pc$ increases or the skewness coefficient of lifetime distribution becomes large for most shift sizes. EWMA, CNN and LSTM charts with CEV have better detection efficiency than EWMA, CNN and LSTM charts with CM in these cases of $Pc=0.2$ for all ${a}_{0}$ values and gamma parameters (${a}_{0}$ = 4, ${b}_{0}$ = 1) for all $Pc$ values.

**Table 6:** SS ARL values of six control charts with $\pi = 1000$.

$Pc$	$\delta$	($a_{0}$, $b_{0}$) = (1, 1)						($a_{0}$, $b_{0}$) = (2, 1)						($a_{0}$, $b_{0}$) = (4, 1)					
		EWMA		CNN		LSTM		EWMA		CNN		LSTM		EWMA		CNN		LSTM	
		CEV	CM	CEV	CM	CEV	CM	CEV	CM	CEV	CM	CEV	CM	CEV	CM	CEV	CM	CEV	CM
$$\lambda = 0.1$$																			
0.2	0.8	**15.52**	17.85	21.59	21.86	32.63	95.62	**9.97**	11.83	12.40	1717	15.15	28.89	**6.69**	9.65	7.38	15.54	11.29	17.41
	0.7	8.78	9.73	**7.38**	8.80	12.53	41.08	6.54	6.36	**4.38**	5.05	6.55	11.20	4.13	5.60	**3.19**	4.65	4.61	6.24
	0.6	4.99	6.78	**3.71**	4.88	6.51	16.26	3.76	5.16	2.51	**2.28**	3.83	5.78	3.05	3.73	**1.66**	1.96	2.45	2.99
	0.5	3.64	4.16	**2.36**	2.75	3.72	7.92	3.30	3.42	**1.50**	**1.50**	2.29	2.44	2.33	2.79	**1.16**	1.30	1.59	1.55
	0.2	2.38	2.55	1.19	**1.17**	1.29	2.44	1.71	1.30	**1.00**	1.01	1.31	1.14	1.68	1.65	**1.00**	**1.00**	1.02	1.06
0.5	0.8	**10.96**	12.37	15.82	13.60	29.42	20.26	8.62	**8.32**	12.52	12.22	18.40	11.43	6.30	6.16	8.73	**5.82**	12.99	10.61
	0.7	5.48	5.19	5.32	**4.31**	13.53	6.95	4.73	5.16	4.01	**3.01**	8.71	5.07	3.72	3.59	2.58	**2.02**	5.56	3.79
	0.6	3.49	3.55	3.04	**1.86**	7.03	3.60	3.77	3.37	2.09	**1.63**	4.74	2.45	2.79	2.53	1.22	**1.13**	2.59	2.04
	0.5	3.73	3.61	2.05	**1.26**	3.70	2.08	3.39	2.10	1.37	**1.17**	2.65	1.68	2.20	1.91	**1.00**	1.05	1.74	1.39
	0.2	1.68	2.21	1.26	**1.00**	1.27	1.04	1.95	1.92	1.03	**1.00**	1.11	1.01	1.48	1.12	**1.00**	1.02	1.05	**1.00**
0.8	0.8	9.78	9.23	10.03	**8.88**	20.20	18.41	7.46	6.63	8.88	**6.61**	18.83	15.49	5.99	5.81	9.04	**5.48**	13.13	10.45
	0.7	4.78	5.99	4.46	**3.61**	9.51	8.11	5.14	3.68	3.23	**2.42**	8.46	6.18	3.50	3.32	**1.99**	2.00	4.81	4.07
	0.6	4.48	3.96	1.98	**1.51**	4.39	3.39	2.35	2.04	2.36	**1.42**	4.44	2.82	2.39	2.39	**1.03**	1.10	2.65	1.96
	0.5	2.11	2.70	1.38	**1.14**	2.45	1.90	1.37	1.34	1.93	**1.13**	2.59	1.81	1.86	1.85	**1.00**	1.05	1.88	1.41
	0.2	2.58	1.28	**1.00**	**1.00**	1.06	1.01	1.22	2.32	1.01	**1.00**	1.12	1.01	1.18	1.14	**1.00**	1.01	1.01	**1.00**
$$\lambda = 0.2$$																			
0.2	0.8	**15.50**	17.21	18.01	23.91	26.72	130.62	**10.33**	13.69	14.49	20.33	17.60	26.88	**6.69**	9.73	6.84	17.60	10.29	19.03
	0.7	8.08	8.57	**7.30**	8.87	11.48	45.06	5.44	6.99	**4.64**	6.89	7.22	10.25	3.72	5.01	**2.69**	3.39	4.51	7.02
	0.6	5.27	5.50	**3.01**	4.39	6.30	16.46	3.61	4.29	**2.51**	2.98	3.82	4.54	2.64	3.16	**1.26**	1.59	2.29	2.97
	0.5	3.90	3.92	2.25	**2.06**	3.88	9.34	2.77	3.02	**1.60**	2.01	2.68	2.49	2.10	2.38	**1.13**	1.15	1.79	1.97
	0.2	2.07	2.26	1.13	**1.00**	1.23	2.52	1.72	1.72	**1.00**	1.14	1.23	1.08	1.26	1.34	**1.00**	**1.00**	1.04	1.01
0.5	0.8	12.28	**10.34**	14.71	13.20	23.71	19.39	9.19	**8.45**	9.57	9.38	18.80	15.26	6.18	6.00	**5.54**	7.31	13.03	10.84
	0.7	6.54	5.26	4.75	**4.03**	12.07	7.68	4.90	4.11	3.33	**3.08**	8.95	6.33	3.48	3.24	**2.33**	2.42	5.17	4.04
	0.6	4.09	3.36	2.58	**2.01**	5.87	3.58	3.30	2.64	1.88	**1.47**	4.67	3.07	2.43	2.13	**1.43**	1.49	2.58	2.07
	0.5	3.14	2.48	1.42	**1.27**	3.78	1.97	2.48	1.95	1.26	**1.17**	2.71	1.77	1.93	1.65	**1.13**	1.19	1.62	1.26
	0.2	1.84	1.39	**1.00**	**1.00**	1.30	1.02	1.59	1.15	1.02	**1.00**	1.07	**1.00**	1.18	1.05	**1.00**	**1.00**	1.02	1.01
0.8	0.8	9.49	8.21	8.83	**7.80**	22.51	21.44	7.69	7.14	**5.95**	8.24	20.79	14.44	5.95	5.52	**5.16**	5.49	13.09	8.68
	0.7	4.85	4.49	3.69	**3.15**	9.23	9.22	4.25	3.78	3.31	**2.56**	8.92	5.16	3.24	3.14	**2.21**	**2.21**	4.70	3.57
	0.6	3.09	2.92	2.43	**1.68**	4.71	4.10	2.76	2.58	1.89	**1.41**	4.49	2.79	2.20	2.08	1.37	**1.28**	2.53	1.87
	0.5	2.27	2.23	1.58	**1.31**	2.71	2.35	2.02	1.96	1.37	**1.17**	2.56	1.62	1.69	1.63	**1.04**	1.05	1.61	1.49
	0.2	1.25	1.31	**1.00**	1.01	1.05	**1.00**	1.22	1.13	1.02	**1.00**	1.11	1.01	1.06	1.04	**1.00**	1.03	1.03	**1.00**

Smallest ARL values are in [bold].

Comparing the ARL values of Tables 4 and 6 for ZS ($\pi$ = 0) and $\pi$ = 1000, the EWMA charts with CEV and CM perform worse detection efficiency under ZS condition than under SS condition. In the LSTM charts with CEV and CM, the detection efficiency increases as $\pi$ increases for most shift sizes, but the efficiency of some small shift sizes performs irregular changes. The CNN charts with CEV and CM have more excellent performance under ZS condition than under SS condition for most shift sizes. As $\pi$ increases, the detection ability of the CNN charts with CEV and CM in most shift sizes slightly reduced but, in most cases of $\delta$ = 0.8, the detection efficiency is significantly reduced.

When the mean shifts occur after the process has been running for a long time, CNN chart will be the best choice for gamma type-I censored data unless there is a need to detect tiny shift sizes. The CNN chart with CEV is suitable for the gamma censored data of lower rates or smaller skewness values, and the CNN chart with CM is recommended for monitoring the moderately, and highly censoring gamma data of larger skewness values.

## Real-world case study

A reliability life test for a liquid–crystal display module (LCM) was conducted at a temperature of 70 °C with 80% relative humidity. Based on historical data analysis, the lifetime distribution of an LCM is known to follow a gamma distribution with shape parameter ${a}_{0}=5.72$ and scale parameter ${b}_{0}=0.48$. To save testing time and cost, practitioners use the censoring rate $Pc=0.8$ to conduct the test and the censoring time ${c}_{T}$ is found to be 1.76 h. The skewness coefficient value of this lifetime distribution is 0.35, which approximates a symmetrical distribution, therefore EWMA CEV statistics is selected for monitoring the LCM’s lifetime. The CEV of this lifetime distribution can be calculated as 3.09. According to quality inspection regulations, five units of each batch of LCMs must be randomly sampled to test lifetime values. Because the EWMA chart based on the CNN network has better performance than the EWMA charts and the EWMA chart on the LSTM network, practitioners developed a CNN-based EWMA chart with CEV using $\lambda =0.1$ and in-control ARL = 200. As shown in Fig. 1, practitioners trained a CNN network using the EWMA CEV statistics in Eq. (4) and obtained the optimal threshold value ${\eta }^{*}=-0.186$ for the in-control ARL value of 200.

In line with the above, practitioners tested five units from each batch under conditions of 70 °C and 80% humidity, and halted testing when the test time reached 1.76 h. The EWMA statistics (Eq. (4)) were inputted into the well-trained CNN network to predict the statistic of the next period. Table 7 showed the lifetime data of testing units for 30 batches. In the 101st batch, only one tested unit failed at 1.23 h and other four tested units did not fail. The lifetime of four unfailed units is recorded as CEV. The actual value ${E}_{j}$ in Table 7 can be obtained by using Eq. (4). As shown in Table 7, the EWMA CEV statistic of the 101st batch was 2.75, so the practitioners inputted 2.75 into the well-trained CNN network, which outputted the predicted value of 2.82 for the 102nd batch. After the 102nd batch was produced and the life tests of the five units reached the termination time of 1.76 h, the actual value ${E}_{j}$ of this batch was determined to be 2.75 by using Eq. (4) and the error value of this batch was − 0.07.

**Table 7:** Predicted and error values of the CNN network for the LCM lifetime test.

No. of batch	Row data (lifetime of failed units)	Actual value $E_{j}$	Predicted value $\widehat{{E_{j} }}$	Error value $E_{j} - \widehat{{E_{j} }}$
101	1.23	2.75		
102	1.30	2.75	2.82	− 0.07
103	1.34, 1.63	2.72	2.75	− 0.03
104	0.97, 1.61, 1.65	2.65	2.59	0.06
105	No failed unit	2.70	2.64	0.06
106	1.37	2.70	2.10	0.60
107	1.66	2.71	2.70	0.01
108	1.17	2.71	2.72	− 0.01
109	1.33	2.71	2.72	− 0.01
110	0.85, 0.92, 1.54	2.63	2.45	0.18
111	1.58	2.65	2.67	− 0.02
112	1.30, 1.40, 1.41	2.59	2.11	0.48
113	1.60, 1.73	2.58	1.88	0.70
114	No failed unit	2.63	2.62	0.01
115	1.47, 1.62	2.62	2.47	0.15
116	1.58	2.63	2.68	− 0.05
117	0.65, 1.56	2.60	2.62	− 0.02
118	No failed unit	2.65	2.73	− 0.08
119	0.28, 1.29	2.60	2.76	− 0.16
120	1.63, 1.67	2.59	2.82	− 0.23
121	0.28, 0.77, 0.95, 1.21, 1.39	2.42	2.62	− 0.20
122	0.54, 1.05, 1.20, 1.55	2.33	2.99	− 0.66
123	0.86, 1.50	2.33	2.67	− 0.34
124	1.06, 1.23, 1.51	2.30	2.65	− 0.35
125	0.51, 0.84, 1.19, 1.19, 1.70	2.18	3.09	− 0.91
126	0.37, 0.72, 1.17	2.13	2.66	− 0.53
127	0.42, 0.79, 0.80, 0.99, 1.00	1.99	2.19	− 0.20
128	0.51, 1.07, 1.31, 1.64	1.95	2.25	− 0.30
129	0.94, 1.18, 1.54	1.95	2.73	− 0.78
130	1.04, 1.17, 1.45	1.95	2.07	− 0.12

**Figure 3:**
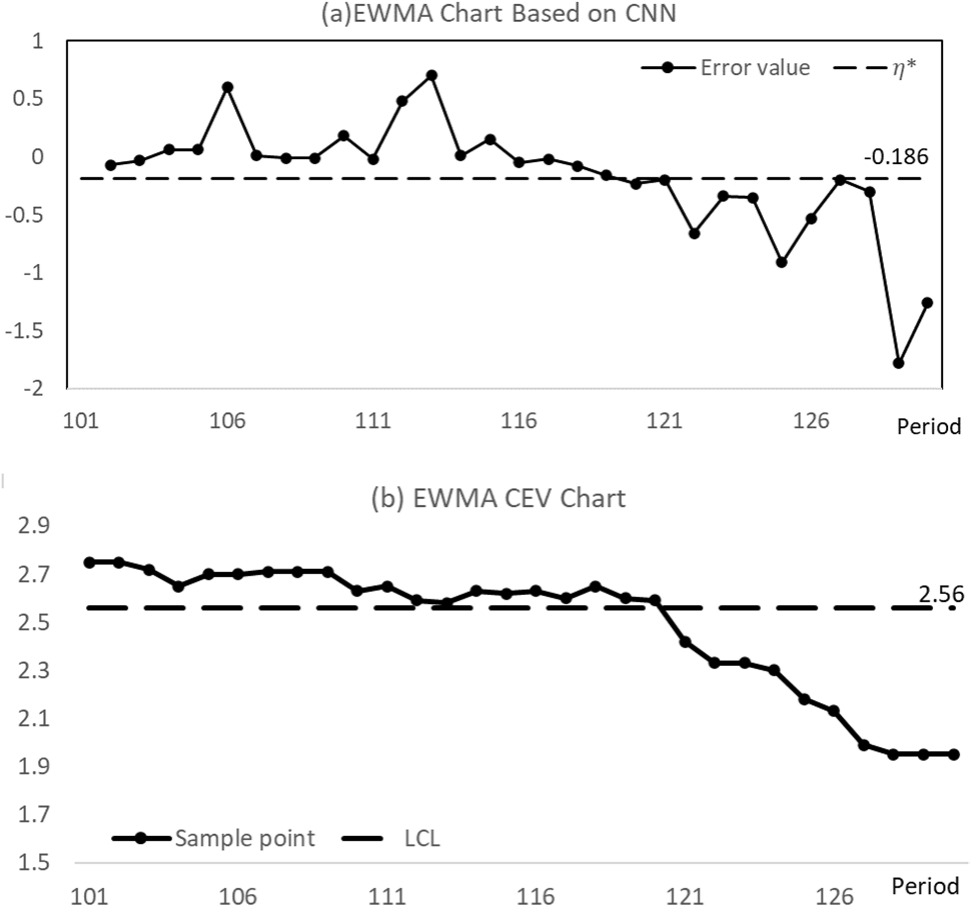
Control charts for monitoring the LCM’s censored data.

In Table 7, other error values were obtained with the same method. Figure 3a plots these error values for the CNN-based control chart. It can be seen that the error values of batches 119–130 were below the ${\eta }^{*}$ value, and so this chart indicates the variation at the 119th batch. Figure 3b also shows the EWMA CEV chart with LCL = 2.56 and its in-control ARL value is approximately 200. The EWMA CEV chart signals the variation at the 121st batch, detecting the same variation more slowly than the CNN-based EWMA chart with CEV.

## Conclusions

The combination of deep learning methods and control charts has greatly improved the efficiency of process monitoring. However, poor efficiency in the monitoring of high type-I censored data using control charts is a challenge for practitioners. This study proposed a control chart based on deep learning methods with EWMA CEV and CM statistics to detect the mean lifetime reduction for gamma type-I censored data. The ZS and SS ARL values of the proposed charts were also measured. Comparing the ZS and SS ARL values of the EWMA chart and the two EWMA charts based on deep learning methods with CEV and CM, CNN-based EWMA chart outperforms other control charts under ZS condition. For SS condition, the EWMA charts based on CNN with CEV and CM outperformed the other charts for various skewness coefficient values and censoring rates for most shift sizes. The EWMA charts with CEV and CM was slightly better than the CNN-based EWMA charts with CEV and CM for a few tiny shift sizes. The EWMA charts based on LSTM with CEV and CM consistently had the worst performance under ZS and SS conditions. In addition, a real-world case study showed that the CNN-based EWMA chart detected mean lifetime reduction more efficiently than the traditional EWMA CEV chart.

For the gamma censored data of lower rates or smaller skewness coefficient values, the CNN-based EWMA chart with CEV is the best choice, and the CNN-based EWMA chart with CM is recommended monitoring the moderately, and highly censoring data of heavily skewed gamma distribution. Future work could extend current approaches to combine CUSUM CEV and CM statistics with deep learning methods to monitor the censored data with normal or non-normal distributions. In addition, there are opportunities to combine multiple deep learning methods to build control charts.

### Supplementary Information

Supplementary Information.

## Data Availability

The datasets generated and/or analyzed during the current study are not publicly available due there are still some industrial projects in progress but are available from the corresponding author on reasonable request.
