# Supplementary material for: Monitoring gamma type-I censored data using an exponentially weighted moving average control chart based on deep learning networks
Source: Sci Rep. 2024 Mar 18;14:6458. doi: 10.1038/s41598-024-56884-8 (PMC10948809; doi:10.1038/s41598-024-56884-8)
Supplement: Supplementary file 1 — Supplementary Information. [file 41598_2024_56884_MOESM1_ESM.docx]

**Supplementary. The CEV and CM of a gamma distribution**

The probability density function $f_{Ga}\left( u \right)$ of the gamma distribution with $a$ and $b$ can be expressed as:

$f_{Ga}\left( u \right)=\frac{u^{a-1}e^{-\frac{u}{b}}}{b^{a}\Gamma\left( a \right)}$,

where $\Gamma\left( \cdot\right)$ is a gamma function. The partial expectation of a gamma distribution with respect to a threshold $y$ is defined as:

$g\left( y \right)=\int_{y}^{\infty} u\times\frac{u^{a-1}e^{-\frac{u}{b}}}{b^{a}\Gamma\left( a \right)}du=\int_{y}^{\infty} \frac{u^{\left( a+1 \right)-1}e^{-\frac{u}{b}}}{b^{a}\Gamma\left( a \right)}du=ab\int_{y}^{\infty} \frac{u^{\left( a+1 \right)-1}e^{-\frac{u}{b}}}{b^{a+1}\Gamma\left( a+1 \right)}du=ab\left[ 1-\int_{0}^{y} \frac{u^{\left( a+1 \right)-1}e^{-\frac{u}{b}}}{b^{a+1}\Gamma\left( a+1 \right)}du \right]$. (A.1)

It can be observed that $\int_{0}^{y} \frac{u^{\left( a+1 \right)-1}e^{-\frac{u}{b}}}{b^{a+1}\Gamma\left( a+1 \right)}du$ is a CDF of the gamma distribution with shape parameter $a+1$ and scale parameter $b$. Therefore, Eq. (A.1) can be rewritten as:

$g\left( y \right)=ab\left[ 1-F_{Ga}\left( u=y | a+1,b \right) \right]$.

The CEV of the gamma distribution is:

$E\left( U | u>y \right)=\frac{\int_{y}^{\infty} u\times f_{Ga}\left( u \right)du}{\int_{y}^{\infty} f_{Ga}\left( u \right)du}=\frac{g\left( y \right)}{1-\int_{0}^{y} f_{Ga}\left( u \right)du}=\frac{ab\left[ 1-F_{Ga}\left( u=y | a+1,b \right) \right]}{1-F_{Ga}\left( u=y | a,b \right)}$.

The CM’s function of the gamma distribution can be represented as:

$M\left( U | u>y \right)=\frac{\int_{0}^{CM} \frac{u^{a-1}e^{-\frac{u}{b}}}{b^{a}\Gamma\left( a \right)}du}{1-F_{Ga}\left( u=y | a,b \right)}=0.5$ (A.2)

The CM can be obtained by solving Eq.(A.2) and the CM of the gamma distribution is:

$CM=F_{Ga}^{-1}\left( 0.5-0.5F_{Ga}\left( u=y | a,b \right) | a,b \right)$.
